# Trends in treatment delays for patients with acute ST-elevation myocardial infarction treated with primary percutaneous coronary intervention

**DOI:** 10.1186/1471-2261-14-115

**Published:** 2014-09-10

**Authors:** Salla Helve, Juho Viikilä, Mika Laine, Jyrki Lilleberg, Ilkka Tierala, Tuomo Nieminen

**Affiliations:** Heart and Lung Center, Cardiology, Helsinki University Central Hospital, P.O. Box 340, FI-00029 Helsinki, Finland; Department of Internal Medicine, Hyvinkää Hospital, Hyvinkää, Finland; Department of Internal Medicine, South Karelia Central Hospital, Lappeenranta, Finland

**Keywords:** Primary percutaneous coronary intervention, Prognosis, ST-segment elevation myocardial infarction, Treatment delays

## Abstract

**Background:**

Treatment delay is an important prognostic factor for patients with acute ST-elevation myocardial infarction (STEMI) treated with primary percutaneous coronary intervention (PCI). We aimed to determine recent trends in these delays and factors associated with longer delays.

**Methods:**

We compared two datasets collected in Helsinki University Central Hospital in 2007–2008 (HUS-STEMI I) and 2011–2012 (HUS-STEMI II), a total of 500 patients treated with primary PCI within 12 hours of the onset of symptoms.

**Results:**

Delays of the emergency medical system (EMS) were longer in HUS-STEMI I than II (medians 81 vs. 67 min, respectively, p < 0.001). Although door-to-balloon times were longer in the later dataset (33 vs. 48 min, p < 0.001) most of the patients (75.3% vs. 62.8%, respectively, p = 0.010) were treated within the recommendation (<60 min) of the European Society of Cardiology (ESC). In HUS-STEMI II, patient arrival at the hospital during off-hours was associated with longer door-to-balloon time (40 and 57.5 min, p = 0.001) and system delay (111 and 127 min, p = 0.009). However, in HUS-STEMI I, arrival time did not impact the delays. Longer system delay was associated with higher mortality rates.

**Conclusions:**

Though the delays inside the hospital have increased they are still mostly within the ESC guidelines. Still, only about half of the patients are treated within a system delay of recommended two hours. Albeit our results are good in comparison with previous studies, further efforts for decreasing the delays particularly within the EMS should be established.

## Background

Total ischemic time is a significant predictor of outcome in patients with acute ST-elevation myocardial infarction (STEMI) [1–3]. Timely reperfusion therapy reduces mortality of patients with STEMI [1, 4–7]. According to international guidelines, primary percutaneous coronary intervention (PCI) is the primary choice of reperfusion therapy if PCI can be performed by an experienced team and within two hours of first medical contact (FMC) [8, 9]. In addition, the overall risk of the patient should be considered when choosing the reperfusion method [10]. Delay between the onset of symptoms and reperfusion therapy reflects the total ischemic time which is why the delay should be reduced as much as possible. However, reperfusion therapy inside the time limits of the international guidelines is seldom achieved in a majority of hospitals [11–13]. Although some earlier studies have reported significant decreases in door-to-balloon times over years, most of the latest studies have only shown limited improvement in treatment delays [11, 12, 14–16]. Previous studies have also indicated that the delays are longer during off-hours than during regular office hours [15, 17, 18].

The objective of this study was to determine the delays for STEMI patients treated with primary PCI in the Helsinki University Central Hospital (HUCH), providing 24/7 PCI-service and being responsible for all primary PCIs in the hospital district of Helsinki and Uusimaa (HUS) in southern Finland. We compared two different datasets, HUS-STEMI I collected in 2007–2008 and HUS-STEMI II in 2011–2012, in order to determine whether there has been any improvement in delays within the last years. In addition, we assessed the variation in the delays between regular and off-hours. Finally, we determined whether longer delays had an effect on the patient outcome.

## Methods

### Data collection

The STEMI patients for the earlier dataset, HUS-STEMI I, were enrolled between June 13, 2007 and June 12, 2008 in the HUS area [19]. Patients were included if they had STEMI and lived permanently in the HUS district. They also had to give a written consent. The criteria for STEMI were symptoms of acute myocardial ischemia and ECG showing at least 2 mm ST-elevation (1.5 mm for women) in at least two of the leads V1-V3 or at least 1 mm ST-elevation in other two adjacent leads (in leads V4-6, V8, V4R, I, aVL, II, III and aVF) or a new left bundle branch block (LBBB). To ensure complete coverage hospital files were also searched to find all additional STEMI patients treated in the HUS area during this time period. The patients who had not been recruited during their hospital stay were contacted later by letters and phone calls. The HUS-STEMI I dataset also included 176 patients treated with fibrinolysis and 78 patients who did not receive any primary reperfusion therapy; these were excluded from the present study.

The other dataset, HUS-STEMI II, comprises of all the STEMI patients treated with primary PCI in HUCH between January 1, 2011 and April 30, 2012. Patients were collected prospectively to a local STEMI-registry to determine the safety and efficacy of a new antithrombotic drug combination, enoxaparine-prasugrel-bivalirudin, introduced in the local STEMI treatment guidelines in the HUS area in autumn 2010. To be included patients had to meet the same criteria for STEMI as in the earlier dataset and be treated with primary PCI within 12 hours from the onset of pain. Patients treated with fibrinolysis were not included in the data. Eighty-one patients were excluded from the present study because they did not live permanently in the HUS area.

The primary PCI patients in HUS-STEMI I received aspirin 250 mg p.o, enoxaparine 30 mg i.v, abciximab bolus 0.25 mg/kg i.v. (or alternatively eptifibatide or tirofiban) and clopidogrel 600 mg p.o. According to the new local guidelines, the patients in the HUS-STEMI II dataset were to receive aspirin 250 mg p.o, enoxaparine 30 mg i.v. and prasugrel 60 mg p.o. upon FMC, and an infusion of bivalirudin in the course of PCI.

The final cohorts included 194 patients from HUS-STEMI I and 306 patients from HUS-STEMI II. Regardless of their differences, the two datasets are comparable. They both contain all STEMI patients treated with primary PCI in HUCH during the data collection period. In addition, the registration of data points was essentially similar.

The study was performed in accordance with the Declaration of Helsinki. The collection of HUS-STEMI I dataset was approved by the ethical board of the HUCH district, and the patients gave the informed consent to participate in the study. The HUS-STEMI II is a registry study, which does not need an ethical approval from the above-mentioned board. However, we received the appropriate approval needed for the registry study as well as the overall approval from our institution to perform the study.

### Guidelines for delays and definitions for time points

Patient delay is the delay from the onset of symptoms to FMC. The time from FMC to the arrival at hospital constitutes the delay of the emergency medical system (EMS). The system delay refers to the sum of the EMS delay and door-to-balloon time within the hospital. The PCI time point was defined as the first balloon inflation. The total ischemic time is the sum of the patient delay and the system delay. We concentrated on the system delay and its components because it is more easily affected by organizational changes such as changes in EMS or hospital strategies. Patient delay can also be affected by recall bias meaning that a patient might be uncertain about the time of the onset of symptoms. According to the latest guidelines stated by the European Society of Cardiology (ESC) the system delay for primary PCI should be less than 90 minutes and for high-risk patients (early presenters within 2 hours or large anterior infarcts) less than 60 minutes. Door-to-balloon time in PCI-capable hospitals should be less than 60 minutes [8].

The time points for the assessment of delays used in this study were the onset of symptoms, first diagnostic ECG (considered as FMC), arrival at PCI providing HUCH hospital (Meilahti), the beginning of angiography and PCI. From these parameters, the patient delay, the delay of EMS, door-to-balloon time, the system delay and the overall delay were calculated.

The arrival time at the hospital was divided to groups based on whether patient arrived during regular or off-hours in order to determine whether there was variation between the time groups. The definition for off-hours used in this study was weekdays between 4 pm and 7 am, weekends and national holidays.

The patient outcomes measured in this study were 90-day all-cause mortality and major adverse cardiac event (MACE) within 90 days of the initial STEMI. The composite of MACE consisted of cardiovascular death, non-fatal myocardial infarction, ischemic or hemorrhagic stroke and unplanned new target vessel or non-target vessel revascularization. Myocardial infarction was defined according to current international guidelines [20]. Stroke was defined as any focal neurological deficit of ischemic or hemorrhagic origin lasting for longer than 24 hours.

### Statistical analysis

Continuous data was expressed as median with interquartile range (IQR) and categorical data as percentages. Percentage of patients treated within the time limits recommended by ESC was calculated. Mann–Whitney U and χ^2^ tests were used for continuous and categorical variables, respectively. A linear regression analysis was used to evaluate the significance of factors possibly related to longer treatment delays. These factors included patient baseline characteristics, prior cardiovascular diseases, Killip class, hospital arrival time and patient related delay. In HUS-STEMI II we also analyzed whether there was a correlation between the delays and adherence to the new local guidelines (enoxaparine, prasugrel and bivalirudine). The delays and relationships were compared between the two datasets. A logistic regression analysis was used for binary covariates such as endpoints. P < 0.05 was considered statistically significant. Statistical analysis was performed with SPSS 15.0 (SPSS, Inc., Chicago, IL).

## Results

A total of 500 patients were included in the analysis (Table 1). HUS-STEMI I had more patients with prior diagnosed hypertension or prior acute myocardial infarction (AMI). Otherwise the baseline characteristics for the datasets were similar. In addition, if each dataset was divided into groups based on hospital arrival in regular compared to off-hours, the two groups were similar with one exception: there were more patients with a previous stroke during off-hours in HUS-STEMI I.

**Table 1:** **Baseline characteristics for both datasets**

	HUS-STEMI I	HUS-STEMI II	
	n = 194	n = 306	
	%	%	p
Mean age, mean (SD) years	64.8 (12.9)	64.3 (14.0)	0.920
Age > 75 years	23.7	26.1	0.542
Male sex	64.9	69.0	0.352
Diabetes	15.5	19.6	0.240
Current smoker	35.6	39.0	0.449
Hypertension	63.4	53.3	0.026
Dyslipidemia	44.8	41.5	0.462
Renal dysfunction	1.5	4.2	0.094
AMI	18.6	10.8	0.014
Stroke	8.8	6.5	0.354
CABG	4.1	5.2	0.573
PCI	9.8	13.4	0.227
ASA	23.2	26.8	0.367
P2Y12 inhibitor	2.1	3.9	0.250
Warfarin	9.3	8.8	0.863
Anterior infarct	46.8	53.6	0.143
Killip class 2--4	12.4	18.0	0.096
Admission at regular hours	41.5	40.8	0.888

Values are percentages with the exception of age.

*AMI*, acute myocardial infarction; *ASA*, acetosalicylic acid; *CABG*, coronary artery bypass graft; *PCI*, percutaneous coronary intervention.

### Changes in delays

**Figure 1:**
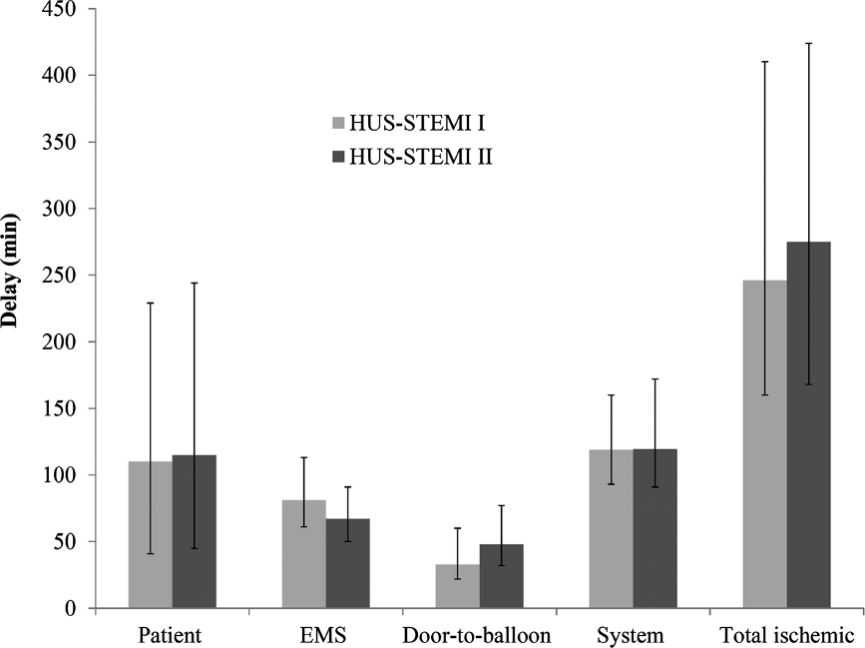
**Median delays (min) with inter-quartile ranges.**

The median EMS delay was longer in HUS-STEMI I than II (81 vs. 67 min, respectively, p < 0.001) (Figure 1). However, the median door-to-balloon time was longer in HUS-STEMI II than I (48 vs. 33 min, respectively, p < 0.001) with total system delays being virtually the same in both datasets (119.5 vs. 119 min, respectively, p = 0.549). There were no significant changes in the patient delays or the total ischemic times, although the delays were numerically slightly longer in HUS-STEMI II.

### Guideline adherence

Recommendations for treatment delays were quite poorly achieved in both HUS-STEMI I and II (Table 2). System delay was less than 90 minutes in only approximately one fourth of the cases. System delay of less than 120 minutes was achieved in a half of the cases; the differences between datasets were not statistically significant. Regarding the achievement of the target time frame of 60 minutes for EMS delay, a clear improvement was seen between the datasets. In HUS-STEMI II 41.0% of patients achieved the target time frame as opposed to 24.7% in HUS-STEMI I. The delays inside the hospital were more often within the recommended time limits (<60 min) in both datasets (Table 2).

**Table 2:** **Guideline adherence: proportion of the delays within the recommended time limits**

Delay	HUS-STEMI I	HUS-STEMI II	
	%	%	p
Patient delay <120 min	55.8	50.8	0.290
EMS delay <60 min	24.7	41.0	<0.001
Door-to-balloon time <60 min	75.3	62.8	0.010
System delay <90 min	22.0	25.0	0.499
System delay <120 min	52.0	50.4	0.761

Time limit recommendations by the European Society of Cardiology (ESC).

*EMS*, emergency medical system.

### Factors associated with delays

In HUS-STEMI I, previous myocardial infarction was associated with shorter EMS delay (Table 3) and previous stroke with longer EMS delay. In addition, if patient related delay was more than 2 hours, the system delay was also longer.

**Table 3:** **Association of patient characteristics to the delays in multivariable linear regression**

			HUS-STEMI I						HUS-STEMI II			
	EMS delay		Door-to-balloon		System delay		EMS delay		Door-to-balloon		System delay	
	Beta	p	Beta	p	Beta	p	Beta	p	Beta	p	Beta	p
Age	0.082	0.394	0.132	0.235	0.139	0.184	-0.007	0.915	-0.028	0.705	-0.039	0.596
Sex	-0.048	0.564	-0.073	0.445	-0.034	0.709	-0.036	0.555	0.010	0.884	0.008	0.899
Smoking	0.095	0.308	0.112	0.295	0.169	0.096	-0.085	0.192	0.019	0.788	-0.050	0.489
Post-MI	-0.198	0.025	0.058	0.566	-0.163	0.091	0.089	0.166	0.047	0.497	0.090	0.193
Post-stroke	0.184	0.030	-0.059	0.545	0.164	0.073	-0.072	0.250	-0.066	0.331	-0.089	0.188
Diabetes	0.152	0.058	0.070	0.459	0.134	0.134	-0.064	0.316	0.003	0.966	-0.003	0.963
Hypertension	-0.048	0.565	-0.002	0.987	-0.046	0.612	0.037	0.586	-0.076	0.295	-0.084	0.248
Dyslipidemia	-0.034	0.686	0.097	0.317	0.032	0.725	-0.012	0.851	0.174	0.016	0.149	0.038
Anterior infarct	-0.092	0.235	-0.028	0.753	-0.026	0.761	0.047	0.433	-0.014	0.831	0.026	0.689
Killip class 2--4	-0.126	0.116	-0.056	0.541	-0.084	0.327	-0.052	0.404	0.141	0.039	0.073	0.278
Regular hours	-0.102	0.202	-0.003	0.974	-0.097	0.260	-0.011	0.851	-0.204	0.002	-0.167	0.010
Patient related delay > 120 min	0.215	0.101	0.059	0.702	0.321	0.027	0.159	0.099	0.017	0.877	0.063	0.556
New treatment protocol							-0.178	0.003	-0.089	0.174	-0.18	0.006

The new treatment protocol in the HUS-STEMI II dataset refers to the use of antithrombotic drug combination enoxaparine-prasugrel-bivalirudin. *MI*, myocardial infarction.

In HUS-STEMI II, patient arrival at hospital during off-hours was strongly associated with longer system delay and door-to-balloon time (Tables 3 and 4). Patients treated according to the new antithrombotic medication protocol had shorter system delays and EMS delays. Treatment according to new guidelines had no effect on the door-to-balloon time. In addition, Killip class 2–4, when admitted to the hospital, was associated with longer door-to-balloon time in adjusted but not in unadjusted logistic regression.

**Table 4:** **Median delays (inter-quartile range) in regular vs. off-hours**

	HUS-STEMI I			HUS-STEMI II		
	Regular hours	Off-hours		Regular hours	Off-hours	
	n = 72	n = 108	p	n = 123	n = 178	p
Patient delay	113 (41–246)	109 (42–221)	0.972	117 (38–243)	110.5 (48–246)	0.612
EMS delay	83 (60–112)	80 (62–121)	0.669	64 (47–89)	68.5 (52–94)	0.270
Door-to-balloon time	37 (25–63)	31 (21–60)	0.464	40 (29–61)	57.5 (34–87)	0.001
System delay	120.5 (92–152)	118.5 (92–174)	0.945	111 (87–166)	127 (105–182)	0.009
Total ischemic time	227.5 (161–398)	255 (159–429)	0.597	263.5 (150–407)	290 (189–439)	0.064

*EMS*, emergency medical system.

The EMS delay was significantly longer in HUS-STEMI I than II during both on- and off-hours. On the other hand, the door-to-balloon time was longer in HUS-STEMI II during off-hours.

### Treatment delays and prognosis

There was no significant difference in the incidence of MACE or death within 90 days between the datasets. The occurrence of MACE was numerically slightly smaller in HUS-STEMI I than II but this was not statistically significant (11.4% and 14.9%, respectively, p = 0.252). Mortality in HUS-STEMI I and II was 10.5% and 10.8%, respectively (p = 0.897).

Longer door-to-balloon time (>60 min) and longer system delay (>90 min) were the only time delay factors that had any effect on the incidence of MACE within 90 days after the STEMI. In unadjusted analysis, 7.9% of the patients with a door-to-balloon time less than 60 minutes had a MACE within 90 days of the STEMI compared to 15.7% of the patients with longer than 60 min door-to-balloon time (p = 0.018). The median door-to-balloon time for patients who had a MACE within 90 days was 60 minutes as opposed to 41 minutes in those without MACE (p = 0.028). Of the patients treated within the system delay of 90 minutes 2.2% had a MACE within 90 days compared to 13.1% for the patients with system delay over 90 minutes (p = 0.003). However, only the association between the system delay and 90-day MACE was statistically significant in regression analysis adjusted to baseline characteristics.

In unadjusted analysis, age (p < 0.001), previous AMI (p = 0.005), previous stroke (p = 0.029), diabetes (p = 0.001), Killip class 2–4 (p < 0.001) were other factors that had a link with 90-day MACE. When adjusted to baseline characteristics, only diabetes and Killip 2–4 were associated with higher incidence of 90-day MACE along with system delay.

Regarding the 90-day mortality, patient related delay was the only time delay factor that had a link with the mortality: in the surviving patient group the median patient related delay was 116 minutes as opposed to 67.5 minutes in the other group (p = 0.044). Yet, this correlation was not statistically significant when adjusted to baseline characteristics.

Other factors associated with 90-day mortality when analyzed individually were age (p < 0.001), sex (p = 0.042), previous AMI (p = 0.017), previous stroke (p < 0.001), diabetes (p = 0.001) and Killip class 2–4 (p < 0.001). When adjusted to baseline characteristics, only age (p = 0.001), previous stroke (p = 0.027), diabetes (p = 0.011) and Killip class 2–4 (p < 0.000) were linked with higher 90-day mortality.

## Discussion

### The delays

The unexpected finding in this study was that door-to-balloon times were longer in 2011–2012 than 2007–2008. On the other hand, the EMS delays had become shorter in the course of years, although still being essentially longer than the in-hospital delays. As the system delay consists of the EMS delay and the delay inside the hospital, the related changes obliterated each other and the system delays between the compared datasets were similar.

Even though the in-hospital delay was longer in the latter than in the earlier dataset, both groups achieved the guidelines for door-to-balloon times quite well and the local delays are short in international comparison. Although being 15 minutes longer than in the earlier dataset, the median door-to-balloon time of 48 minutes in HUS-STEMI II is clearly within the recommendation of 60 minutes [8]. As many as 75.3% of patients were treated within 60 minutes in HUS-STEMI I, while the same target was reached in 62.8% of the HUS-STEMI II patients (Table 2). Some earlier studies have reported significant improvement in the door-to-balloon times over time but the results are from the early years of primary PCI, when the decrease in delays was more expectable [15, 16]. More recent studies have shown less improvement in door-to-balloon times over time. In a study from a multinational registry GRACE, the door-to-balloon times remained practically unchanged (75–84 minutes) during the study period from 1999 to 2006 with an exception of the year 1999 when the median door-to-balloon time was 99 minutes [11]. However, a recent study of the large CathPCI registry from the USA showed significant improvement in door-to-balloon times over the study period: median door-to-balloon times decreased from 83 min to 67 min and the percentage of patients treated within 90 minutes increased from 60% to 83% from 2005 to 2009 [21].

The adherence to recommended time limits was poorer in other delays than door-to-balloon. The EMS delay has shrunk over time, but still only 41% of patients were transported to the PCI hospital in 60 minutes. The distances in the HUS area are relatively short with about two thirds of population being gathered in the city of Helsinki and its suburbs with up to 20–30 km distance to the Meilahti hospital of HUCH. The longest distances to the Meilahti hospital are about 100 km.

The median system delay in both datasets was a minute short of two hours. Only 22% in HUS-STEMI I and 25% in HUS-STEMI II were treated within 90 minutes (Table 2). The results from a US report from the National Registry of Myocardial Infarction from 1999 to 2002 were quite similar showing no substantial improvement in average treatment times: the percentage of patients with a system delay less than 90 minutes was 35% in 1999 and increased by 2% in 2002 [12]. Another study that described the delays in 30 European countries had median system delays of 60 to 177 minutes [13]. The system delay of our study is placed just in the middle of these European results. In our study, the majority of system delay is composed of the EMS delay, thus the efforts to decrease the delays should be focused on EMS.

Patient related delay was quite long in both datasets, medians being 110 vs. 115 minutes in former vs. later dataset. This forms almost a half of the total ischemic time, which is why attempts for reducing this delay should be established. Public campaigns have been effective for the recognition of the common symptoms of a stroke [22, 23]; a similar strategy would most probably increase the awareness of the symptoms and shorten the delays in the context of AMI.

In a Swedish study, which analyzed the trends in the treatment of STEMI patients from 1996 to 2007, the total ischemic times first increased from 185 min to 216 min and then decreased again to 203 min over the study period. Even the present study demonstrated an increasing tendency for the delays over time. The total ischemic times in our study were considerably longer, 246 min and 275 min in HUS-STEMI I and II, respectively. However, further comparison between these studies is not possible because the Swedish study does not categorize the different components of the total ischemic time [14].

### Factors associated with delays

Presentation to the hospital during off-hours seemed to be strongly associated with longer delays in HUS-STEMI II. The difference in door-to-balloon times between regular and off-hours was 17.5 minutes and, correspondingly, in system delays 16 minutes. During off-hours the angiography team is on-call so it could be expected that the delays inside the hospital are longer than in the regular hours. The results from previous studies assessing the variation in treatment delays between regular and off-hours are quite similar to our results from HUS-STEMI II. Reported door-to-balloon times have been 21–25 minutes longer during off- than on-hours [15, 16]. Interestingly, hospital presentation time did not affect the delays in the HUS-STEMI I dataset. The difference in the impact of off-hours presentation on the in-hospital delays between the datasets might reflect the delegation of night-time initial STEMI consultation calls from interventional cardiologist to the physician in the coronary care unit in HUCH; this reorganization occurred after the HUS-STEMI I period. The interventional cardiologists participating in the off-hours PCI procedures were virtually the same during both study periods and all of them live within 30 minutes from the hospital.

Patients treated according to the new local guidelines of antithrombotic medication also had shorter system and EMS delays. Treatment according to the new guidelines did not affect the door-to-balloon time. Therefore, the new protocol does not seem to cause any additional delay either inside the hospital or in the EMS.

In HUS-STEMI I, patients who waited for more than 2 hours before contacting the EMS had longer system delays. This may reflect less specific symptoms in these patients, which would lengthen the delays in different steps.

### Endpoints

The 90-day mortality and the occurrence of major adverse cardiac event (MACE) in 90 days were similar in the two datasets. This is expected based on the fact that there were no significant differences in the system delays or total ischemic times. The small numerical decrease in the mortality may reflect development in other treatment for the STEMI patients. Longer system delay was associated with higher mortality rates in our study which is in accordance with previous studies [1, 3, 7].

Medication given prior and/or during the PCI may influence the mortality [24]. In our data, treatment according to new guidelines did not affect 90-day MACE or mortality.

### Strengths and limitations of this study

Both datasets contained all STEMI patients treated with primary PCI in HUCH during the data collection period. Thus, the study population represents the true STEMI population of the HUS area. An observational study cannot prove causality between the factors associated with delays or the endpoints. Furthermore, there were so few endpoints that the correlation between the delays and endpoints could not be analyzed reliably.

## Conclusions

Door-to-balloon times have become longer but are still relatively well within the limits of the European guidelines. The EMS delays have become shorter but are still long in comparison to door-to-balloon times. Arrival at hospital during off-hours was the only factor clearly associated with longer delays inside the hospital suggesting that changes in the hospital strategies during off-hours should be considered.

## References

[1] De Luca G, Suryapranata H, Ottervanger JP, Antman EM (2004). Time delay to treatment and mortality in primary angioplasty for acute myocardial infarction, every minute counts. Circulation.

[2] Brodie BR, Webb J, Cox DA, Qureshi M, Kalynych A, Turco M, Schultheiss HP, Dulas D, Rutherford B, Antoniucci D, Stuckey T, Krucoff M, Gibbons R, Lansky A, Na Y, Mehran R, Stone GW, EMERALD investigators (2007). Impact of time to treatment on myocardial reperfusion and infarct size with primary percutaneous coronary intervention for acute myocardial infarction (from the EMERALD trial). Am J Cardiol.

[3] Shiomi H, Nakagawa Y, Morimoto T, Furukawa Y, Nakano A, Shirai S, Taniguchi R, Yamaji K, Nagao K, Suyama T, Mitsuoka H, Araki M, Takashima H, Mizoguchi T, Eisawa H, Sugiyama S, Kimura T, CREDO-Kyoto AMI investigators (2012). Association of onset to balloon and door to balloon time with long term clinical outcome in patients with ST elevation acute myocardial infarction having primary percutaneous coronary intervention: observational study. BMJ.

[4] Cannon CP, Gibson CM, Lambrew CT, Shoultz DA, Levy D, French WJ, Gore JM, Weaver WD, Rogers WJ, Tiefenbrunn AJ (2000). Relationship of symptom-onset-to-balloon time and door-to-balloon time with mortality in patients undergoing angioplasty for acute myocardial infarction. JAMA.

[5] Brodie BR, Gersh BJ, Stuckey T, Witzenbichler B, Guagliumi G, Peruga JZ, Dudek D, Grines CL, Cox D, Parise H, Prasad A, Lansky AJ, Mehran R, Stone GW (2010). When is door to balloon time critical? Analysis from the HORIZONS-AMI (Harmonizing outcomes with revascularization and stents in acute myocardial infarction) and CADILLAC (Controlled abciximab and device investigation to lower late angioplasty complications) trials. J Am Coll Cardiol.

[6] McNamara RL, Wang Y, Herrin J, Curtis JP, Bradley EH, Magid DJ, Peterson ED, Blaney M, Frederick PD, Krumholtz HM, NRMI Investigators (2006). Effect of door-to-balloon time on mortality in patients with ST-segment elevation myocardial infarction. J Am Coll Cardiol.

[7] Terkelsen CJ, Sørensen JT, Maeng M, Jensen LO, Tilsted H, Trautner S, Vach W, Johnsen SP, Thuesen L, Lassen JF (2010). System delay and mortality among patients with STEMI treated with primary percutaneous coronay intervention. JAMA.

[8] Steg PG, James SK, Atar D, Badano LP, Lundqvist CB, Borger MA, Di Mario C, Dickstein K, Ducrocq G, Fernandez-Aviles F, Gershlick AH, Giannuzzi P, Halvorsen S, Huber K, Juni P, Kastrati A, Knuuti J, Lenzen MJ, Mahaffey KW, Valgimigli M, Van-‘t Hof A, Widimsky P, Zahger D (2012). ESC Guidelines for the management of acute myocardial infarction in patients presenting with ST-segment elevation: The Task Force on the management of ST-segment elevation acute myocardial infarction of the European Society of Cardiology (ESC). Eur Heart J.

[9] Boersma E, the Primary Coronary Angioplasty vs. Thrombolysis (PCAT)-2 Trialists’ Collaborative Group (2006). Does time matter? A pooled analysis of randomized clinical trials comparing primary percutaneous coronary intervention and in-hospital fibrinolysis in acute myocardial infarction patients. Eur Heart J.

[10] Pinto DS, Kirtane AJ, Nallamothu BK, Murphy SA, Cohen DJ, Laham RJ, Cutlip DE, Bates ER, Frederick PD, Miller DP, Carrozza JP, Antman EM, Cannon CP, Gibson CM (2006). Hospital delays in reperfusion for ST-elevation myocardial infarction: implications when selecting a reperfusion strategy. Circulation.

[11] Eagle KA, Nallamothu BK, Mehta RH, Granger CB, Steg PG, Van de Werf F, López-Sendón J, Goodman SG, Quill A, Fox KA, Global Registry of Acute Coronary Events (GRACE) Investigators (2008). Trends in acute reperfusion therapy for ST-segment elevation myocardial infarction from 1999 to 2006: we are getting better but we have got a long way to go. Eur Heart J.

[12] McNamara RL, Herrin J, Bradley EH, Portnay EL, Curtis JP, Wang Y, Magid DJ, Blaney M, Krumholz HM, NRMI Investigators (2006). Hospital improvement in time to reperfusion in patients with acute myocardial infarction, 1999–2002. J Am Coll Cardiol.

[13] Widimsky P, Wijns W, Fajadet J, de Belder M, Knot J, Aaberge L, Andrikopoulos G, Baz JA, Betriu A, Claeys M, Danchin N, Djambazov S, Erne P, Hartikainen J, Huber K, Kala P, Klinceva M, Kristensen SD, Ludman P, Ferre JM, Merkely B, Milicic D, Morais J, Noc M, Opolski G, Ostojic M, Radovanovic D, De Servi S, Stenestrand U, Studencan M (2012). European Association for Percutaneous Cardiovascular Interventions: Reperfusion therapy for ST elevation acute myocardial infarction in Europe: description of the current situation in 30 countries. Eur Heart J.

[14] Jernberg T, Johanson P, Held C, Svennblad B, Lindbäck J, Wallentin L, SWEDEHEART/RIKS-HIA (2011). Association between adoption of evidence-based treatment and survival for patients with ST-elevation myocardial infarction. JAMA.

[15] Angeja BG, Gibson CM, Chin R, Frederick PD, Every NR, Ross AM, Stone GW, Barron HV, Participants in the National Registry of Myocardial Infarction 2–3 (2002). Predictors of door-to-balloon delay in primary angioplasty. Am J Cardiol.

[16] Gibson CM, Pride YB, Frederic PD, Pollack CV, Canto JG, Tiefenbrunn AJ, Weaver WD, Lambrew CT, French WJ, Peterson ED, Rogers WJ (2008). Trends in reperfusion strategies, door-to-needle and door-to-balloon times, and in-hospital mortality among patients with ST-segment elevation myocardial infarction enrolled in the National Registry of Myocardial Infarction from 1990 to 2006. Am Heart J.

[17] Magid DJ, Wang Y, Herrin J, McNamara RL, Bradley EH, Curtis JP, Pollack CV, French WJ, Blaney ME, Krumholz HM (2005). Relationship between time of day, day of week, timeliness of reperfusion and in hospital mortality for patients with acute ST-segment elevation myocardial infarction. JAMA.

[18] Blakenship JC, Skelding KA, Scott TD, Berger PB, Parise H, Brodie BR, Dudek D, Möckel M, Lansky AJ, Mehran R (2010). Predictors of reperfusion delay in patients with acute myocardial infarction undergoing primary percutaneous coronary intervention from the HORIZONS-AMI trial. Am J Cardiol.

[19] Viikilä J, Lilleberg J, Tierala I, Syvänne M, Kupari M, Salomaa V, Nieminen MS, the HUS-STEMI Investigators (2013). Outcome up to one year following different reperfusion strategies in acute ST-segment elevation myocardial infarction: The Helsinki-Uusimaa Hospital District registry of ST-Elevation Acute Myocardial Infarction (HUS-STEMI). Eur Heart J.

[20] Thygesen K, Alpert JS, Jaffe AS, Simoons ML, Chaitman BR, White HD, Writing Group on the Joint ESC/ACCF/AHA/WHF Task Force for the Universal Definition of Myocardial Infarction (2012). Third universal definition of myocardial infarction. Eur Heart J.

[21] Menees DS, Peterson ED, Wang Y, Curtis JP, Messenger JC, Rumsfeld JS, Gurm HS (2013). Door-to-balloon time and mortality among patients undergoing primary PCI. N Engl J Med.

[22] Robinson TG, Reid A, Haunton VJ, Wilson A, Naylor AR (2013). The face arm speech test: does it encourage rapid recognition of important stroke warning symptoms?. Emerg Med J.

[23] Silver FL, Rubini F, Black D, Hodgson CS (2003). Advertising strategies to increase public knowledge of the warning signs of stroke. Stroke.

[24] Prati F, Petronio S, Van Boven AJ, Tendera M, De Luca L, de Belder MA, Galassi AR, Imola F, Montalescot G, Peruga JZ, Barnathan ES, Ellis S, Savonitto S, FINESSE-ANGIO substudy investigators (2010). Evaluation of infarct-related coronary artery patency and microcirculatory function after facilitated percutaneous primary coronary angioplasty: the FINESSE-ANGIO (Facilitated Intervention with Enhanced Reperfusion Speed to Stop Events-Angiographic) study. JACC Cardiovasc Interv.

[CR25] The pre-publication history for this paper can be accessed here:http://www.biomedcentral.com/1471-2261/14/115/prepub

